# Mortality trends associated with hypertension and atrial fibrillation: A CDC WONDER data analysis

**DOI:** 10.1016/j.gloepi.2025.100217

**Published:** 2025-09-26

**Authors:** Saad Khan, Usama Idrees, Safa Nasir, Fatima Naveed, Aqsa Munir, Muhammad Junaid Iqbal, Rizwan Ahmad, Muhammad Ubaid Hussain, Fathimathul henna, Khansha Saeed, Amin ul Haq, Ali Ahmed

**Affiliations:** a Saidu Medical College, Department of Internal Medicine, Saidu Shareef, Mingora City, District Swat, KPK 19130, Pakistan; b Khawaja Muhammad Safdar Medical College, Department of Internal Medicine, Commissioner Road, City Sialkot, Punjab 51310, Pakistan; c Agha Khan University Hospital, Department of Internal Medicine, Stadium Road, City Karachi, Sindh 75350, Pakistan; d Rawal Institute of Health Sciences, Department of Internal Medicine, Almehrban Road, Madina Town, Tarlai Kalan, City Islamabad, Pakistan, 45550; e Dow Medical College, Department of Internal Medicine, Mission Road, Nanakwara, City Karachi, Sindh 74800, Pakistan; f University of Urbino, Department of Biomolecular Sciences, Via Saffi, 2, Urbino PU 61029, Italy; g Nawaz Sharif Medical College, Department of Internal Medicine, Near University of Gujrat, City Gujrat, Punjab 50700, Pakistan; h Dubai Medical College for Girls, Department of Internal Medicine, Muhaisnah 1, Dubai, UAE; i Rashid Latif Medical College, Department of Allied Surgery, Central Park Housing Scheme, City Lahore, Punjab 54600, Pakistan; j Saidu Teaching Hospital, Department of Internal Medicine, Saidu Shareef, Mingora City, District Swat, KPK 19130, Pakistan; k Division of Infectious Diseases and Global Public Health, School of Medicine, University of California San Diego (UCSD), 9500 Gilman Drive, La Jolla, CA, 92093, USA

**Keywords:** Hypertension, Atrial fibrillation, Mortality, United states, CDC WONDER

## Abstract

**Background:**

Hypertension (HTN) and atrial fibrillation (AF) are prevalent cardiovascular disorders that significantly increase the risk of serious complications such as stroke and heart failure, leading to elevated mortality rates. Despite the established relationship between HTN and AF, there is a lack of comprehensive evidence on mortality trends and disparities across various demographic groups in the United States.

**Objectives:**

This study aims to analyze the nationwide mortality trends due to HTN-AF from 1999 to 2020 and to identify disparities across different demographics. The goals include understanding the impact of HTN-AF on public health and informing targeted screening and therapeutic strategies.

**Methods:**

Data on mortality figures related to HTN and AF were obtained from the Centers for Disease Control and Prevention's (CDC) Wide-Ranging Online Data for Epidemiologic Research (WONDER) database. The analysis included age-adjusted mortality rates (AAMR) and crude mortality rates (CMR) stratified by gender, age, race/ethnicity, and geographic regions. The Joinpoint software was used to analyze temporal trends in age-adjusted mortality rate (AAMR). Data were obtained from publicly available multiple causes of death records via the CDC WONDER database.

**Results:**

From 1999 to 2020, the AAMR due to hypertension-attributable factors rose sharply from 2.89 to 23.98 per 100,000 (APC: 4.8 %). Males had higher AAMRs than females, with Black or African American populations seeing the steepest increases. Regionally, the West had the highest AAMR, and rural areas experienced the most significant rise, with micropolitan areas showing the highest APC.

**Conclusion:**

HTN-AF mortality has been increasing steadily across all genders, races, and regions. The study underscores the importance of improving healthcare policies, bridging coverage gaps, and enhancing education and awareness to curb these alarming trends. Addressing the disparities in healthcare access and promoting cardiovascular health initiatives are crucial for reducing HTN-AF-related mortality.

## Introduction

Hypertension (HTN) and atrial fibrillation (AF) are two common cardiovascular disorders that often co-exist. These conditions substantially increase the risk of serious complications, such as stroke and heart failure, which in turn significantly elevate overall mortality [1,2]. Hypertension is characterized by persistently elevated blood pressure and affects approximately 47 % of adults in the United States [3]. Over time, hypertension triggers structural and electrical changes in the heart, thereby increasing the likelihood of atrial fibrillation (AF) onset. AF, in turn, promotes blood stagnation and clot formation, which elevates the risk of thromboembolic events such as embolic stroke. Additionally, the irregular heart rhythm associated with atrial fibrillation can impair cardiac function and lead to heart failure [1]. This vicious cycle significantly increases the risk of stroke, heart failure, and sudden cardiac death, thereby contributing to the high mortality rates [2].

There is an established relationship between hypertension and atrial fibrillation. Evidence from the Framingham Heart Study suggests that hypertension elevates the risk of progressing to AF by roughly 50 % in both men and women [4]. Another study has demonstrated that aggressive blood pressure control can decrease the incidence and frequency of AF [5]. Despite the established relationship between hypertension (HTN) and atrial fibrillation (AF), there is limited literature exploring mortality-related events associated with HTN and AF across the United States. Although a few studies have investigated mortality trends attributed to hypertension (HTN) and atrial fibrillation (AF) alone, the cumulative health risks posed by these conditions remain uninvestigated. (A1). Highlighting these gaps is essential to understanding the broader impact of hypertension and atrial fibrillation (HTN-AF) on public health. It is also critical to develop targeted treatments that could reduce mortality and morbidity in vulnerable populations [6]. Approaching these literature gaps could provide more crucial insights and guidelines for enhancing patient outcomes and decreasing the mortality associated with these conditions [7,8,9].

Our study aims to assess the nationwide mortality trends due to HTN-AF during the past two decades (1999–2020) and gain critical insights into the disparities across different demographics. This would help design better screening and therapeutic strategies for vulnerable populations. Additionally, it may guide the judicious allocation of healthcare resources towards areas showing high mortality rates.

## Methods

### Data source

The data for the current study, which involved mortality related to hypertension (HTN) and atrial fibrillation (AF) from 1999 to 2020 across the United States, was obtained from the Centers for Disease Control and Prevention (CDC) Wide-Ranging Online Data for Epidemiologic Research (WONDER) [10]. The data was obtained by exploring publicly available multiple causes of death records using The International Classification of Diseases, Tenth Revision (ICD-10) codes: I10 for essential hypertension and I48 for atrial fibrillation, respectively [11]. The CDC WONDER database is based on mortality records compiled from death certificates across the United States. It has been widely utilized in previous studies [12]. No institutional review board approval was obtained for the current research, as it used publicly available de-identified data from CDC WONDER [10].

### Data abstraction

The data for HTN-AF-related deaths from 1999 to 2020 across the entire United States was abstracted for the population aged 25 and above. The mortality rates were analyzed by gender (male, female), age (25–34, 35–44, 45–54, 55–64, 65–74, 75–84 and ≥ 85), race/ethnicity (Hispanic, Non-Hispanic White, Non-Hispanic Black, Non-Hispanic Asian or Pacific Islander, Non-Hispanic American Indian/Native American), US census regions (Northeast, Midwest, South, and West according to the US Census Bureau definitions) [13]. The geographical data was further grouped per the National Center for Health Statistics' (NCHS) Urban–Rural Classification Scheme for Counties into urban, large metropolitan areas with a population equal to or more than 1 million, medium/small metropolitan areas with a population between 50,000 and 999,999, and rural areas with a population less than 50,000 [14].

### Statistical analysis

The HTN-AF-related mortalities were analyzed using both crude and age-adjusted mortality rates (AAMRs), expressed per 100,000 population. Crude mortality rates (CMRs) were calculated by dividing the number of deaths in each year by the corresponding U.S. population. Age-adjusted mortality rates were standardized to the 2000 U.S. standard population [15]. To assess national trends in HTN-AF-related mortality, we used Joinpoint regression analysis via the Joinpoint software (version 5.2.0, National Cancer Institute) [16]. Joinpoint regression identifies statistically significant changes in trend over time by fitting segmented linear models to the log-transformed AAMR values. Each joinpoint represents a time point where a significant change in the slope occurs. The model accounts for heteroscedasticity and autocorrelation through built-in Monte Carlo permutation methods, and provides Annual Percent Changes (APCs) with 95 % confidence intervals (CIs) and *p*-values. To further evaluate temporal trends, we applied linear regression models on log-transformed AAMR and CMR values, assuming an identity link function. We clarify that this approach differs from traditional log-linear models used in generalized linear modeling. Where applicable, we assessed key assumptions of linear regression and *t*-tests, including normality of residuals, homogeneity of variance, and linearity, using diagnostic plots and formal tests (e.g., Shapiro-Wilk, Levene's test). A p-value less than 0.05 was considered statistically significant. As our regression model used log-transformed AAMR values as the response variable, the beta coefficients reflect changes on the logarithmic scale. If exponentiated, these coefficients would represent **geometric mean ratios** rather than arithmetic changes. We did not use a log-linear model with a log link and untransformed response variable, and our findings are not interpreted in terms of incidence rate ratios or relative risk.

## Results

From 1999 to 2020, a total of 693,959 people died due to hypertension with atrial fibrillation (HTN-AF) across the United States. The AAMR due to HTN-AF was 2.89 in 1999 per 100,000 for the entire USA population, which escalated to 23.98 in 2020. The HTN-AF-related deaths increased significantly from 1999 to 2020 (APC; 4.80, 95 % CI; 5.7 to 14.5). (Supplemental Fig. 1).

### Sex

**Fig. 1:**
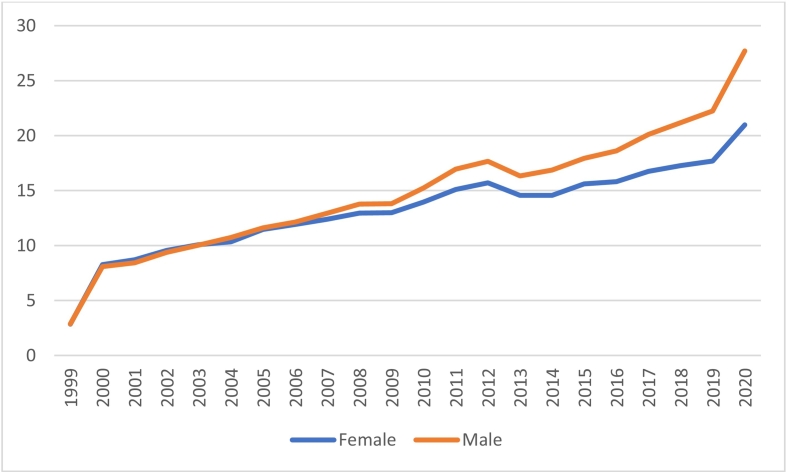
Hypertension and atrial fibrillation- related Age-Adjusted Mortality Rates per 100,000, Stratified by gender in the United States, 1999 to 2020.

Males were reported to have higher mortalities than females throughout the study period. The overall age-adjusted mortality rate (AAMR) for males was 15.68, whereas, for females, it was 13.51. The AAMR for females increased consistently from 2.84 in 1999 to 20.97 in 2020 (APC; 4.1, 95 % CI; 3.2–5.1). However, no joinpoint was obtained for females. On the other hand, trends in mortalities of male gender exhibited one joinpoint. The AAMR for males initially increased significantly from 2.87 in 1999 to 8.42 in 2001 (APC: 49.9 %, 95 % CI: −6.1 to 139.5). The wide confidence interval likely reflects the small number of deaths and the short time span in this early segment, which increases statistical variability and limits the precision of the APC estimate. The AAMR continued to increase more steadily over the following years, reaching 27.70 in 2020 (APC: 5.21 %, 95 % CI: 4.5 to 5.8). (Fig. 1, Supplement Tables 2 and 3).

### Race

The AAMR for American Indian or Alaska Native (AI/AN) and Hispanic or Latino (H/L) increased consistently from 1999 to 2020 without showing any significant change in trend, thus yielding no joinpoint. For AI/AN, the AAMR transitioned from 3.821 in 2000 to 15.0 in 2020. For H/L, the AAMR shifted from 1.634 in 1999 to 15.013 in 2020.

The remaining races demonstrated prominent changes in the mortality trends, each giving two joinpoints. Asian or Pacific Islanders (A/PI) had an AAMR of 1.526 in 1999, which increased to 9.75 in 2011 (APC; 4.86, 95 % CI; 2.4 to 7.2)., after which it dropped to 7.79 in 2014 (APC;-8.22, 95 % CI;-27.4 to 16.1). Later, the AAMR for A/PI started to increase and reached 12.73 in 2020 (APC; 6.99, 95 % CI; 3.6 to 10.4).

The AAMR for White cohort initially rose from 2.95 in 1999 to 16.54 in 2011 (APC;5.89, 95 % CI;4.8 to 6.9) followed by a second relatively slower rise up to 18.08 in 2016 (APC;1.55, 95 % CI;-1.8 to 5.0) next to which the AMRR skyrocketed to 25.27 in 2020 (APC;8.34, 95 % CI;5.2 to 11.5).

**Fig. 2:**
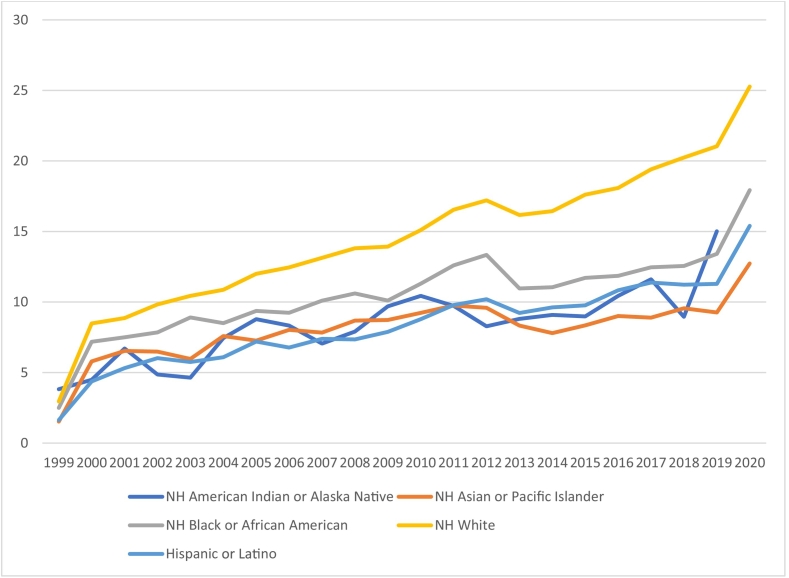
Hypertension and atrial fibrillation -related Age-Adjusted Mortality Rates per 100,000, Stratified by race in the United States, 1999 to 2020.

The Black or African American (B/AA) experienced an increase in AAMR from 2.49 in 1999 to 12.59 in 2011 (APC;4.67, 95 % CI;3.2 to 6.1), followed by a period of no significant change until 2018 when the AAMR was 12.55 (APC;-0.06, 95 % CI;-2.6 to 2.5). The AAMR escalated afterward, up to 17.92 at the end of the study duration in 2020 (APC;20.97, 95 % CI;6.5 to 37.3). (Fig. 2, Supplement Tables 2 and 4).

### Region

The West manifested the highest overall AAMR (16.59), while the South experienced the lowest AAMR (14.12) among all the regions. All four regions demonstrated only one joinpoint each, indicating a consistent transition in AAMR devoid of any unremarkably prominent changes. Additionally, AAMRs for all regions increased significantly from 1999 to 2020.

For the Northeast, the AAMR jumped from 2.98 to 20.71 (APC;4.32 95 % CI;3.3 to 5.2).

The Midwest's AAMR escalated from 3.27 to 25.4 (APC;4.78, 95 % CI;3.8 to 5.7).

The South region exhibited the greatest APC (5.33) with a 95 % CI of 4.4 to 6.3, while its AAMR changed from 2.18 to 24.23 (APC;5.40, 95 % CI;4.4 to 6.3).

**Fig. 3:**
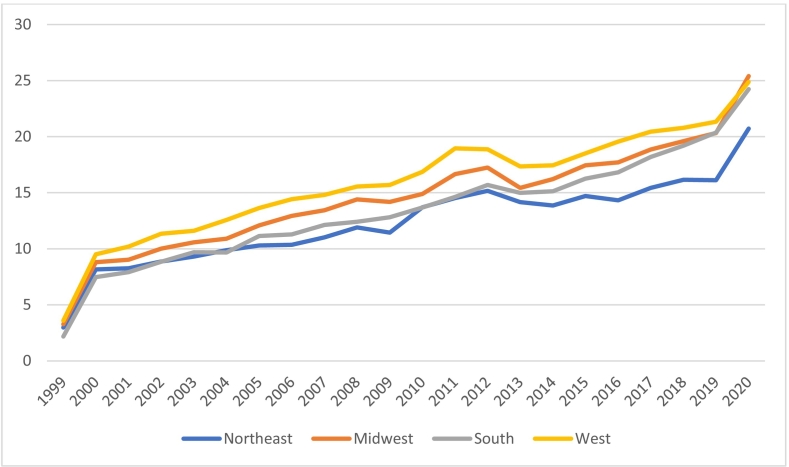
Hypertension and atrial fibrillation-related Deaths, Stratified by region Adults in the United States, 1999 to 2020.

The West's AAMR increased from 3.59 to 24.88, with the lowest APC among all regions (APC;4.22, 95 % CI;3.3 to 5.1). (Fig. 3, Supplement Tables 1 and 2).

### Urbanization

The highest overall AAMR (16.47) was reported in the Micropolitan (MP) areas, while the lowest (13.42) was recorded in the large fringe metro (LFM). None of the urbanization-based strata showed any joint point, suggesting that mortality trends do not have any prominent transition. The AAMR for all segments increased consistently and in almost a similar fashion from 1999 to 2020.

Large Central Metro (LCM) initially noted an AAMR of 2.91 in 1999, which increased to 21.14 in 2020. From 1999 to 2020, the APC was 4.23, with a 95 % confidence interval of 3.2 to 5.19.

The slightly less urbanized Large Fringe Metros had an AAMR of 2.85 in 1999, which increased to 22.15 in 2020(APC; 4.76, 95 % CI;3.8 to 5.6).

The AAMR for the Medium metro (MM) also increased from 2.99 in 1999 to 24.45 in 2020 (APC;4.63, 95 % CI;3.7 to 5.5).

In the case of Small metros (SM), the AAMR shifted from 3.10 in 1999 to 26.73 in 2020 (APC;5.11, 95 % CI;4.1 to 6.0).

The relatively less urbanized Micropolitan areas had an AAMR of 3.04 in 1999, which later increased to 29.91 in 2020 (APC;5.43, 95 % CI;4.4 to 6.3).

**Fig. 4:**
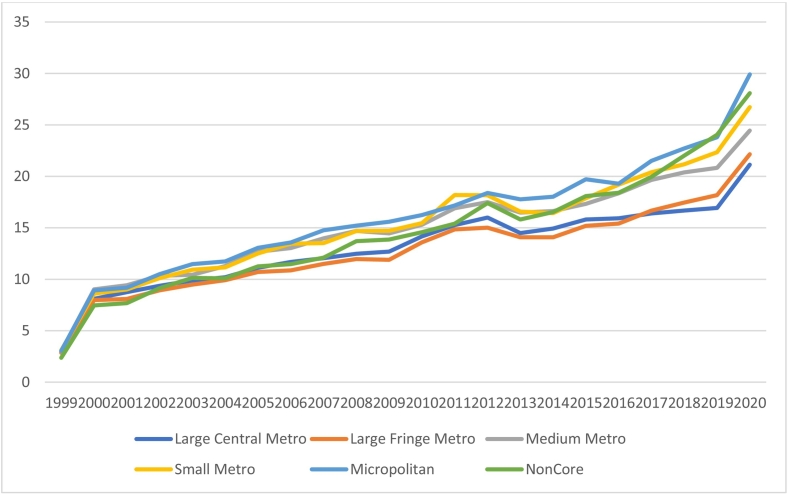
Hypertension and atrial fibrillation -related Age-Adjusted Mortality Rates per 100,000, Stratified by Urban-Rural Classification in the United States, 1999 to 2020.

The relatively rural Non-core (NC) segments manifested the highest APC of 6.22, with a 95 % confidence interval of 5.2 to 7.1. The AAMR, in this case, increased from the initial 2.37 in 1999 to 28.09 in 2020. (Fig. 4, Supplement Tables 2 and 5).

## Discussion

Hypertension is a well-established independent risk factor for atrial fibrillation, one of the most prevalent and serious cardiac rhythm disorders. Atrial fibrillation is associated with significant morbidity and mortality in the general population. Notably, hypertension alone accounts for approximately 20 % of new-onset atrial fibrillation cases [2]. AFib prevalence increased fourfold in the age-adjusted period between 1958 and 1967 and 1998–2007, and hypertension increased from 26.4 % in 2000 to 31.1 % in 2010 [17,18] (Supplemental Fig. 1). This parallel rise underscores the closely intertwined epidemiological trends between these two conditions. Given this alarming pattern, the significance of studying the impact of hypertension on atrial fibrillation-related mortality has become increasingly critical.

**Fig. 5:**
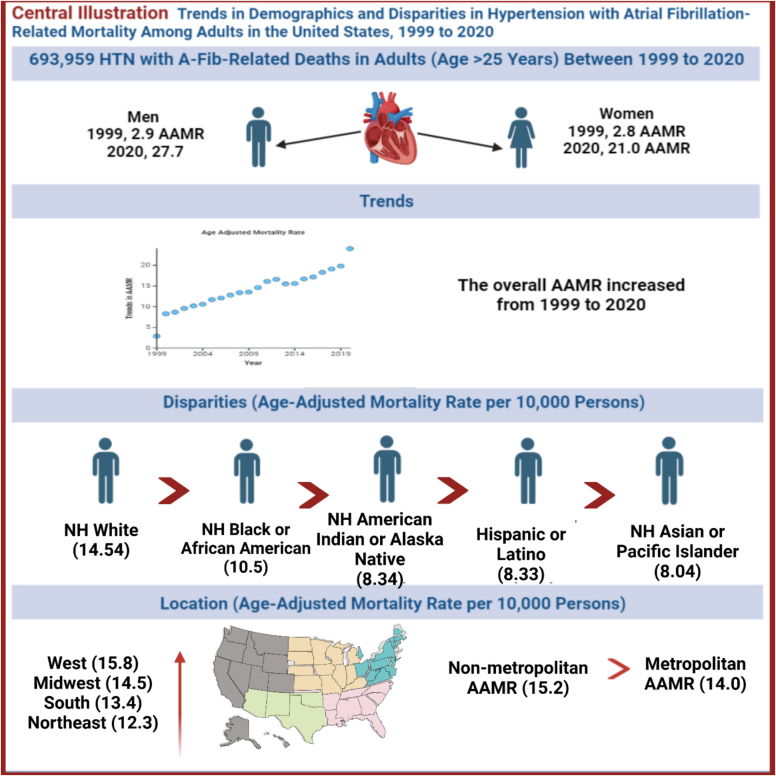
Central illustration.

The analysis of hypertension and atrial fibrillation (AFib) mortality trends from 1999 to 2020 reveals significant disparities across gender, race, region, and urbanization levels. Overall, the Age-Adjusted Mortality Rate (AAMR) for hypertension-atrial fibrillation has consistently increased over the years, reflecting trends similar to other cardiovascular diseases, such as heart failure. [19]Males show a sharper and faster increase than females, while racial groups such as Black and White populations experience alarming recent surges. Regionally, the South leads with the highest rates, while rural areas face steeper increases compared to urban centers, highlighting the effect of limited healthcare access. (Fig. 5 Central Illustration).

Upon gender-based analysis, our study revealed comparable results of HTN-AFIB mortality, with the annual percent change (APC) in the last decade being 5.2 % for men and 4.19 % for women. Similarly, Schnabel observed a fourfold increase in atrial fibrillation (AFib) prevalence over 50 years in men, compared to a threefold increase in women, reinforcing the notion that male gender is a common risk factor for developing AFib [17]. Interestingly, although men exhibited higher mortality rates than women, they also demonstrated a significant decrease in APC from the previous decade. In contrast, women displayed a consistent pattern of increasing trends, raising serious concerns. This discrepancy may be attributed to women presenting with atypical symptoms more frequently than men, which could lead to misdiagnosis [20]. Notably, elderly women are often at a higher risk of stroke- a common and serious consequence of HTN-AFIB [21]. This risk may be further compounded by women's underrepresentation in cardiovascular trials and suboptimal antithrombotic therapy, which results from the underestimation of prognostic scores specifically for women in stroke risk assessment. [22,23]

Furthermore, newer data indicate that when adjusting for factors such as height, women without cardiovascular disease may have a higher risk of atrial fibrillation than men, challenging traditional notions about sex differences in AF risk [24]. This is further supported by evidence indicating that women, especially those with hypertension, may be more predisposed to atrial fibrillation at similar body sizes. This increased risk is attributed to higher levels of atrial fibrosis and a greater likelihood of left atrial dilation or dysfunction, conditions commonly associated with left ventricular hypertrophy, compared to men [25,26].

Increased cardiovascular surveillance, close monitoring, and further research into the biological factors contributing to HTN-AFib in women are essential to developing gender-specific management guidelines. Such guidelines are crucial for addressing the heightened mortality pattern observed in women with HTN-AFib over the years.

Interestingly, while men consistently exhibited higher HTN-AF mortality than women, the annual percent change (APC) in mortality among men declined substantially in the most recent decade compared to the prior one, indicating a slowing of the mortality growth rate rather than an actual decline in deaths. This attenuation in trend may reflect the impact of improved hypertension control, routine cardiovascular screening, and earlier detection and management of atrial fibrillation in male populations [27].

Our analysis of different races confirms that Black and Hispanic populations are at higher risk for AFib-HTN compared to whites, evidenced by the steady AMMR (APC: 0.06 % from 2011 to 1018) for the Black population. Notably, in the Black population, individuals 30 years or younger are disproportionately affected by HTN, leading to earlier disease onset, more severe progression, and a higher overall health burden [28,29]. This is significant given the younger median age of the Black population and the fact that many in this group lack adequate insurance coverage, as insurance programs tend to focus more on the elderly and are disproportionate towards different racial communities [30,31]. The gaps in coverage, particularly after Obamacare-related disenrollment began in 2018, likely contributed to the sudden upward spike in Afib-HTN cases, reflected in a dramatic 348-fold increase in the annual percentage change (from APC: 0.06 % to 20.97 %) [32]. Additionally, rising inflation and economic stressors disproportionately impact the Black community, pushing many closer to the poverty line [33]. Efforts to eliminate the coverage gap and increase medical access are crucial in preventing mortality surges in high-risk populations.

Similarly, despite being high-risk populations, American Indian and Hispanic or Latino groups did not experience a significant change in HTN-AFib mortality rates, with steady AMMRs reported throughout the years. Even though insurance coverage rates have increased in these communities, they remain inadequately covered—much like the Black population—leading to persistent health disparities and unchanged mortality rates [32]. In contrast, the Asian population saw a significant increase in deaths after 2014(APC: −8.22 % to 6.99 %). This rise is likely linked to the Affordable Care Act, which expanded insurance coverage to an ethnic group that was previously extremely underinsured. As a result, more Asians gained access to medical care, leading to increased diagnoses and reporting of HTN-AFib-related deaths [34]. Furthermore, rising immigration from Asian countries, particularly from South Asia, has added to the disease burden in this population, highlighting the need for expanded medical assistance and greater coverage to meet their healthcare needs [35].

The sharp rise in AMMR among the white population since 2016 (APC: 5.89 % to 8.34 %), in addition to challenges faced by the black and Asian population, is alarming and warrants immediate investigation. Notably, this period coincided with a significant decrease in life expectancy in the U.S., largely driven by drug overdoses, suicides, and chronic systemic diseases such as chronic liver disease. [36] The white population was disproportionately impacted, likely due to easier access to prescription opioids, which might have fuelled the opioid epidemic [[36], [37], [38]]. It also likely increased the prevalence or exacerbated the existing AFIB-HTN burden. Psychosocial stressors and economic inflation also likely contribute to this issue, though these relationships require further exploration. [39]

Alongside racial disparities, regional disparities in healthcare outcomes are heavily influenced by local demographics. The South, which is home to a large African American population and historically experiences a higher burden of chronic illnesses, exhibited the steepest increase in HTN-AF mortality trends (APC: 5.40 %) over the study period. Interestingly, despite this rapid rise, the region maintained the lowest overall age-adjusted mortality rate (AAMR: 14.2) by 2020. This paradox may reflect a later onset of the HTN-AF mortality surge in the South, resulting in a sharper recent increase from a lower baseline. It also suggests that while the current burden appears lower, the trajectory is concerning and warrants urgent intervention to prevent further escalation. In contrast, the West exhibits a lower rate of change in mortality (APC: 4.22 %) yet has the highest age-adjusted mortality rate (AAMR) at 16.59. [40]. Similarly, the Northeast, a region where more comprehensive and inclusive health policies have been implemented, exhibited lower APC (4.3 %). [41]

The variability and discrepancy across the regions are alarming. The challenges in the South are exacerbated by the limited implementation of the Affordable Care Act (ACA) in several Southern states, where resistance to adoption—especially in ten states, including Alabama, Florida, Georgia, Kansas, and Mississippi—has left approximately 1.6 million adults without sufficient coverage [40,42,43]. Additionally, issues like residential segregation and limited access to primary care services are more prevalent in the South than in the Northeast or Midwest, exacerbating healthcare inequalities [40,44].

In contrast, the Midwest and Western regions have shown more stable trends in the Annual Percent Change (APC) for AFib-HTN mortality, with APCs of 4.78 % and 4.22 %, respectively. The Northeast also exhibits a similar trend, with an APC of 4.32 %. However, the West stands out with the highest age-adjusted mortality rate (AMMR) at 16.59 over the past two decades.

This could be attributed to its rapid urbanization (88.9 % compared to 75.8 % in the south in 2020) and high rates of immigration, with California being the hub of most immigrants, which create challenges in fund allocation and healthcare infrastructure development [45,46]. While urban areas in the West may have more resources, the accelerated pace of urban growth presents unique challenges for healthcare access and equitable service distribution. Strategic urbanization and paced development are crucial to mitigating these health disparities and associated outcomes.

Finally, our analysis underscores the continuous risk of HTN-AFib mortality in rural U.S. populations. Mortality rates are highest in rural areas (APC: 6.22 % in non-metropolitan areas and APC: 5.43 % in micropolitan areas) and lowest in urban regions(APC:4.23 %). This disparity is likely due to reduced healthcare access and awareness in rural areas, leading to less frequent monitoring and follow-up care.”. [47] Moreover, the limited access to primary care physicians and healthcare services in rural communities further exacerbates this upward mortality trend [33,48]. Addressing this issue requires improving healthcare infrastructure, expanding access to primary care, and promoting cardiovascular health initiatives to reduce future mortality.

While we presented stratified trend analyses by sex, race, region, and urbanization, we acknowledge that these demographic and geographic variables may serve as **potential effect modifiers** of the relationship between broader cardiovascular risk factors and HTN-AF mortality. However, our dataset—based on death certificate data—does not contain individual-level exposures (e.g., smoking, obesity, socioeconomic status) required to directly model such associations. Future research integrating mortality data with individual-level clinical or survey data (e.g., NHANES or Medicare) would be valuable for exploring **causal interactions** and identifying at-risk subgroups with greater precision.

## Conclusion

The study's findings reveal a concerning rise in mortality related to hypertension and atrial fibrillation across all demographics, with marked disparities influenced by gender, race, and geography. This persistent upward trend signals an urgent need for intervention among high-risk groups. These include men, White and Black or African American communities, and those living in rural areas or West or South. They face distinct challenges in accessing healthcare and preventive services. Addressing these disparities requires policies that focus on fair healthcare distribution, increased education on cardiovascular risks, and improved screening and prevention for hypertension and atrial fibrillation. Addressing these essential factors could help decrease the rising mortality rates and improve health outcomes among vulnerable populations. By doing so, we pave the way towards a more equitable framework for cardiovascular health across the United States.(Table 1)

**Table 1:** Public recommendations.

Public health recommendations:
**Rural Healthcare Expansion**: Increase access to cardiovascular care in rural areas through telemedicine, mobile clinics, and provider incentives.
**Gender-Specific Screening**: Train providers on gender-specific symptoms to improve diagnostic accuracy in men with HTN-AF.
**Regional Focus**: Allocate resources to the Southern and Western U.S. for targeted prevention, education, and healthcare access improvements.
**Affordable Medications**: Enhance access to HTN-AF medications through cost-lowering policies and patient assistance programs.
**Community Screening Programs**: By establishing blood pressure and ECG screenings in high-prevalence areas, we can identify HTN-AF early and significantly improve outcomes. This is a reason for optimism in our fight against cardiovascular diseases.
**Community Lifestyle Programs**: Promote exercise, diet, and weight management programs to inspire and empower local communities. These initiatives can significantly reduce HTN-AF risks and improve overall health.
**Emergency Response Training**: Provide first responders and community members training in atrial fibrillation symptom identification and emergency management.

## Limitations

This article has several limitations that warrant consideration. First, as with studies utilizing ICD codes and death certificate data, there is a chance for misclassification or underreporting of hypertension with atrial fibrillation as a cause of death. This could introduce bias. To include a broader population, we utilized multiple cause-of-death data rather than solely the underlying cause of death, which might show a less precise representation of the disease burden. Second, the dataset lacks detailed clinical information, such as blood pressure measurements, electrocardiogram data, and echocardiography findings, which are important for assessing hypertension and atrial fibrillation severity and management. Third, there is no available data on medications or treatment interventions. This limits our ability to evaluate the impact of various therapeutic strategies on patient outcomes. Lastly, the study does not incorporate information on social determinants of health, including socioeconomic status, education, and healthcare access, which could significantly impact disease management and progression in patients with hypertension and atrial fibrillation. One more limitation of CDC WONDER is that it records deaths, not prevalence or incidence of living individuals with HTN-AF. Therefore, the total number of people “still alive with HTN-AF” over 1999–2020 cannot be directly calculated from your current dataset.

## CRediT authorship contribution statement

**Saad Khan:** Writing – review & editing, Writing – original draft, Data curation, Conceptualization. **Usama Idrees:** Writing – review & editing, Writing – original draft, Supervision, Project administration, Data curation. **Safa Nasir:** Writing – review & editing, Writing – original draft, Investigation, Formal analysis. **Fatima Naveed:** Writing – review & editing, Writing – original draft, Visualization, Data curation. **Aqsa Munir:** Writing – review & editing, Writing – original draft, Resources, Data curation. **Muhammad Junaid Iqbal:** Writing – review & editing, Writing – original draft, Supervision. **Rizwan Ahmad:** Writing – review & editing, Writing – original draft, Visualization, Software, Resources. **Muhammad Ubaid Hussain:** Writing – original draft, Methodology, Investigation, Formal analysis. **Fathimathul henna:** Writing – review & editing, Writing – original draft, Visualization. **Amin ul Haq:** Writing – review & editing, Writing – original draft, Supervision. **Ali Ahmed:** Writing – review & editing, Writing – original draft, Supervision.

## Declaration of competing interest

The authors declare that they have no known competing financial interests or personal relationships that could have appeared to influence the work reported in this paper.

## Appendix A Supplementary data

Supplementary material Image 1
