# Supplementary material for: Mortality trends associated with hypertension and atrial fibrillation: A CDC WONDER data analysis
Source: Glob Epidemiol. 2025 Sep 26;10:100217. doi: 10.1016/j.gloepi.2025.100217 (PMC12539261; doi:10.1016/j.gloepi.2025.100217)
Supplement: Supplementary file 1 — Supplementary material [file mmc1.docx]

**Supplemental Files:**

Supplemental Figure 1 Age-Adjusted Mortality Rates per 100,000 for Deaths Related to Hypertension and atrial fibrillation

supplemental Table 1: Hypertension and atrial fibrillation related Deaths, Stratified by region Adults in the United States, 1999 to 2020

Supplemental Table 2: Annual percent change (APC) of Hypertension and atrial fibrillation –related Age-Adjusted Mortality Rates per 10,000 in the United States, 1999 to 2020

supplemental Table 3:  Hypertension and atrial fibrillation related Age-Adjusted Mortality Rates per 100,000, Stratified by gender in the United States, 1999 to 2020

Supplemental Table 4: Hypertension and atrial fibrillation -related deaths and Age-Adjusted Mortality Rates per 100,000, Stratified by race in the United States, 1999 to 2020

Supplemental Table 5: Hypertension and atrial fibrillation -related Age-Adjusted Mortality Rates per 100,000, Stratified by Urban-Rural Classification in the United States, 1999 to 2020

**Supplemental Figure 1:** Age-adjusted mortality rates per 100,000 population for HTN-AF

| **Supplemental Table 1:** Overall Annual Percentage Change (APC) of Hypertension-Atrial fibrillation related Age Adjusted Mortality Rates (AAMRs) per 100,000 in the United States, 1999-2020 | |
| --- | --- |
| **Year Interval** | **APCs (95% CI)** |
| **Overall** |  |
| 1999-2020 | 4.8 (5.7 to 14.5) |
| **Men** |  |
| 1999-2001 | 49.9 (-6.1 to 139.5) |
| 2001-2020 | 5.2 (4.5-5.8) |
| **Women** |  |
| 1999-2020 | 4.1 (3.2 to 5.1) |
| **NH American Indian or Alaska Native** |  |
| 2000-2020 | 4.2 (2.7 to 5.7) |
| **NH Asian or Pacific Islander** |  |
| 2000-2011 | 4.8 (2.4 to 7.2) |
| 2011-2014 | -8.2 (-27.4 to 16.1) |
| 2014-2020 | 6.9 (3.6 to 10.4) |
| **NH Black or African American** |  |
| 2000-2011 | 4.6 (3.2 to 6.1) |
| 2011-2018 | -0.05 (-2.6 to 2.5) |
| 2018-2020 | 20.9 (6.5 to 37.3) |
| **NH White** |  |
| 2000-2011 | 5.8 (4.8 to 6.9) |
| 2011-2016 | 1.5 (-1.8 to 5.0) |
| 2016-2020 | 8.3 (5.2-11.5) |
| **Hispanic or Latino** |  |
| 2000-2020 | 4.8 (4.1 to 5.6) |
| **Northeast** |  |
| 1999-2020 | 4.3 (3.3 to 5.2) |
| **Midwest** |  |
| 1999-2020 | 4.7 (3.8 to 5.7) |
| **South** |  |
| 1999-2020 | 5.3 (4.4 to 6.3) |
| **West** |  |
| 1999-2020 | 4.2 (3.3 to 5.1) |
| **Large Central Metro** |  |
| 1999-2020 | 4.2 (3.2 to 5.2) |
| **Large Fringe Metro** |  |
| 1999-2020 | 4.8 (3.8 to 5.6) |
| **Medium Metro** |  |
| 1999-2020 | 4.6 (3.7 to 5.5) |
| **Small Metro** |  |
| 1999-2020 | 5.1 (4.1 to 6.0) |
| **Micropolitan (Non-Metropolitan)** |  |
| 1999-2020 | 5.4 (4.4 to 6.3) |
| **Noncore (non-metropolitan)** |  |
| 1999-2020 | 6.2 (5.2 to 7.2) |

| **Supplemental Table 2:** Hypertension-Atrial Fibrillation related Age Adjusted Mortality Rate (AAMR) per 100,000, stratified by Gender in the United States, 1999-2020 | | | |
| --- | --- | --- | --- |
| **Year** | **Females**  **AAMR (95%CI)** | **Males**  **AAMR (95%CI)** | **Overall**  **AAMR (95%CI)** |
| 1999 | 2.85(2.75-2.95) | 2.87(2.74-3.01) | 2.89(2.81-2.97) |
| 2000 | 8.27(8.10-8.43) | 8.08(7.86-8.30) | 8.33(8.20-8.47) |
| 2001 | 8.70(8.53-8.87) | 8.42(8.20-8.65) | 8.72(8.58-8.86) |
| 2002 | 9.56(9.38-9.74) | 9.38(9.14-9.61) | 9.63(9.49-9.78) |
| 2003 | 10.08(9.90-10.26) | 10.04(9.80-10.29) | 10.20(10.06-10.35) |
| 2004 | 10.34(10.16-10.52) | 10.73(10.49-10.98) | 10.61(10.47-10.76) |
| 2005 | 11.47(11.28-11.66) | 11.61(11.36-11.81) | 11.70(11.55-11.86) |
| 2006 | 11.92(11.72-12.11) | 12.13(11.87-12.38) | 12.11(11.96-12.27) |
| 2007 | 12.39(12.2-12.59) | 12.94(12.68-13.19) | 12.77(12.61-12.93) |
| 2008 | 12.95(12.76-13.15) | 13.76(13.50-14.03) | 13.44(13.28-13.59) |
| 2009 | 12.98(12.78-13.18) | 13.81(13.55-14.07) | 13.48(13.32-13.63) |
| 2010 | 13.95(13.75-14.16) | 15.24(14.97-15.51) | 14.64(14.47-14.80) |
| 2011 | 15.10(14.89-15.31) | 16.95(16.67-17.23) | 16.01(15.84-16.18) |
| 2012 | 15.70(15.49-15.91) | 17.66(17.38-17.94) | 16.64(16.47-16.81) |
| 2013 | 14.56(14.36-14.76) | 16.33(16.07-16.60) | 15.45(15.29-15.62) |
| 2014 | 14.56(14.36-14.76) | 16.87(16.60-17.13) | 15.64(15.48-15.80) |
| 2015 | 15.61(15.40-15.81) | 17.94(17.67-18.21) | 16.71(16.55-16.88) |
| 2016 | 15.81(15.60-16.01 | 18.61(18.34-18.88) | 17.17(17-17.33) |
| 2017 | 16.75(16.54-16.96) | 20.10(19.82-20.38) | 18.3(18.13-18.47) |
| 2018 | 17.27(17.06-17.47) | 21.17(20.89-21.45) | 19.09(18.92-19.26) |
| 2019 | 17.68(17.48-17.89) | 22.24(21.95-22.52) | 19.77(19.60-19.94) |
| 2020 | 20.98(20.75-21.21) | 27.70(27.39-28.02) | 23.98(23.79-24.17) |

|  | **Supplemental Table 3:** Hypertension-Atrial Fibrillation related deaths and AAMRs per 100,000 stratified by Race in the United States, 1999-2020 | | | |
| --- | --- | --- | --- | --- |
| **Race** | | **Year** | **Total no. of deaths** | **AAMR (95% CI)** |
| American Indian or Alaska Native | | 2000 | 27 | 3.821(2.50-5.60) |
| American Indian or Alaska Native | | 2001 | 36 | 4.49(3.09-6.31) |
| American Indian or Alaska Native | | 2002 | 50 | 6.707(4.93-8.92) |
| American Indian or Alaska Native | | 2003 | 39 | 4.862(3.41-6.73) |
| American Indian or Alaska Native | | 2004 | 39 | 4.642(3.25-6.43) |
| American Indian or Alaska Native | | 2005 | 61 | 7.441(5.65-9.62) |
| American Indian or Alaska Native | | 2006 | 77 | 8.785(6.89-11.05) |
| American Indian or Alaska Native | | 2007 | 80 | 8.332(6.53-10.48) |
| American Indian or Alaska Native | | 2008 | 72 | 7.051(5.45-8.97) |
| American Indian or Alaska Native | | 2009 | 82 | 7.907(6.23-9.90) |
| American Indian or Alaska Native | | 2010 | 106 | 9.691(7.78-11.60) |
| American Indian or Alaska Native | | 2011 | 124 | 10.437(8.54-12.34) |
| American Indian or Alaska Native | | 2012 | 125 | 9.726(7.95-11.50) |
| American Indian or Alaska Native | | 2013 | 118 | 8.277(6.72-9.84) |
| American Indian or Alaska Native | | 2014 | 130 | 8.805(7.23-10.38) |
| American Indian or Alaska Native | | 2015 | 143 | 9.088(7.55-10.63) |
| American Indian or Alaska Native | | 2016 | 152 | 8.977(7.50-10.46) |
| American Indian or Alaska Native | | 2017 | 183 | 10.432(8.87-11.99) |
| American Indian or Alaska Native | | 2018 | 222 | 11.61(10.03-13.19) |
| American Indian or Alaska Native | | 2019 | 187 | 8.948(7.62-10.28) |
| American Indian or Alaska Native | | 2020 | 311 | 15.013(13.30-16.73) |
| Asian or Pacific Islander | | 1999 | 48 | 1.526(1.11-2.04) |
| Asian or Pacific Islander | | 2000 | 210 | 5.794(4.99-6.60) |
| Asian or Pacific Islander | | 2001 | 255 | 6.534(5.71-7.36) |
| Asian or Pacific Islander | | 2002 | 276 | 6.486(5.70-7.27) |
| Asian or Pacific Islander | | 2003 | 264 | 5.963(5.23-6.70) |
| Asian or Pacific Islander | | 2004 | 354 | 7.586(6.78-8.39) |
| Asian or Pacific Islander | | 2005 | 378 | 7.265(6.52-8.01) |
| Asian or Pacific Islander | | 2006 | 433 | 8.026(7.26-8.80) |
| Asian or Pacific Islander | | 2007 | 452 | 7.83(7.10-8.56) |
| Asian or Pacific Islander | | 2008 | 525 | 8.683(7.93-9.44) |
| Asian or Pacific Islander | | 2009 | 567 | 8.724(8.00-9.45) |
| Asian or Pacific Islander | | 2010 | 619 | 9.228(8.49-9.96) |
| Asian or Pacific Islander | | 2011 | 726 | 9.752(9.04-10.47) |
| Asian or Pacific Islander | | 2012 | 765 | 9.592(8.91-10.28) |
| Asian or Pacific Islander | | 2013 | 722 | 8.333(7.72-8.95)) |
| Asian or Pacific Islander | | 2014 | 730 | 7.798(7.23-8.37) |
| Asian or Pacific Islander | | 2015 | 840 | 8.342(7.77-8.91) |
| Asian or Pacific Islander | | 2016 | 953 | 9.01(8.43-9.59) |
| Asian or Pacific Islander | | 2017 | 1010 | 8.889(8.34-9.44) |
| Asian or Pacific Islander | | 2018 | 1146 | 9.557(9.00-10.12) |
| Asian or Pacific Islander | | 2019 | 1178 | 9.261(8.73-9.79) |
| Asian or Pacific Islander | | 2020 | 1707 | 12.735(12.13--13.34) |
| Black or African American | | 1999 | 351 | 2.498(-2.24-2.76) |
| Black or African American | | 2000 | 1021 | 7.186(6.74-7.63) |
| Black or African American | | 2001 | 1076 | 7.502(7.05-7.95) |
| Black or African American | | 2002 | 1145 | 7.844(7.39-8.30) |
| Black or African American | | 2003 | 1310 | 8.908(8.42-9.40) |
| Black or African American | | 2004 | 1287 | 8.504(8.03-8.98) |
| Black or African American | | 2005 | 1444 | 9.366(8.88-9.86) |
| Black or African American | | 2006 | 1467 | 9.239(8.76-9.72) |
| Black or African American | | 2007 | 1638 | 10.097(9.60-10.59) |
| Black or African American | | 2008 | 1765 | 10.607(10.11-11.11) |
| Black or African American | | 2009 | 1750 | 10.106(9.63-10.59) |
| Black or African American | | 2010 | 1987 | 11.297(10.79-11.80) |
| Black or African American | | 2011 | 2318 | 12.595(12.07-13.12) |
| Black or African American | | 2012 | 2555 | 13.339(12.81-13.87) |
| Black or African American | | 2013 | 2196 | 10.96(10.49-11.43) |
| Black or African American | | 2014 | 2286 | 11.051(10.59-11.51) |
| Black or African American | | 2015 | 2522 | 11.714(11.25-12.18) |
| Black or African American | | 2016 | 2644 | 11.853(11.39-12.31) |
| Black or African American | | 2017 | 2877 | 12.464(12.00-12.93) |
| Black or African American | | 2018 | 3010 | 12.556(12.10-13.01) |
| Black or African American | | 2019 | 3320 | 13.411(12.95-13.88) |
| Black or African American | | 2020 | 4622 | 17.929(17.40-18.46) |
| White | | 1999 | 4679 | 2.954(2.87-3.04) |
| White | | 2000 | 13581 | 8.479(8.34-8.62) |
| White | | 2001 | 14403 | 8.863(8.72-9.01) |
| White | | 2002 | 16177 | 9.833(9.68-9.99) |
| White | | 2003 | 17389 | 10.433(10.28-10.59) |
| White | | 2004 | 18365 | 10.871(10.71-11.03) |
| White | | 2005 | 20674 | 12.001(11.84-12.17) |
| White | | 2006 | 21954 | 12.454(12.29-12.62) |
| White | | 2007 | 23574 | 13.131(12.96-13.30) |
| White | | 2008 | 25307 | 13.808(13.64-13.98) |
| White | | 2009 | 25937 | 13.925(13.76-14.10) |
| White | | 2010 | 28652 | 15.098(14.92-15.27) |
| White | | 2011 | 32195 | 16.544(16.36-16.73) |
| White | | 2012 | 34231 | 17.204(17.02-17.39) |
| White | | 2013 | 32777 | 16.165(15.99-16.34) |
| White | | 2014 | 34028 | 16.437(16.26-16.61) |
| White | | 2015 | 37105 | 17.611(17.43-17.79) |
| White | | 2016 | 38773 | 18.084(17.90-18.27) |
| White | | 2017 | 42330 | 19.4(19.21-19.59) |
| White | | 2018 | 45107 | 20.238(20.05-20.43) |
| White | | 2019 | 47652 | 21.039(20.85-21.23) |
| White | | 2020 | 57948 | 25.271(25.06-25.48) |
| Hispanic or Latino | | 1999 | 116 | 1.634(1.33-1.94) |
| Hispanic or Latino | | 2000 | 334 | 4.371(3.89-4.85) |
| Hispanic or Latino | | 2001 | 421 | 5.315(4.80-5.83) |
| Hispanic or Latino | | 2002 | 495 | 6.025(5.48-6.57) |
| Hispanic or Latino | | 2003 | 508 | 5.746(5.23-6.26) |
| Hispanic or Latino | | 2004 | 555 | 6.089(5.57-6.61) |
| Hispanic or Latino | | 2005 | 708 | 7.195(6.65-7.74) |
| Hispanic or Latino | | 2006 | 699 | 6.773(6.26-7.29) |
| Hispanic or Latino | | 2007 | 802 | 7.395(6.87-7.92) |
| Hispanic or Latino | | 2008 | 842 | 7.349(6.85-7.85) |
| Hispanic or Latino | | 2009 | 963 | 7.886(7.38-8.39) |
| Hispanic or Latino | | 2010 | 1104 | 8.783(8.26-9.31) |
| Hispanic or Latino | | 2011 | 1369 | 9.786(9.26-10.31) |
| Hispanic or Latino | | 2012 | 1507 | 10.196(9.68-10.72) |
| Hispanic or Latino | | 2013 | 1456 | 9.222(8.74-9.70) |
| Hispanic or Latino | | 2014 | 1601 | 9.622(9.15-10.10) |
| Hispanic or Latino | | 2015 | 1764 | 9.767(9.30-10.23) |
| Hispanic or Latino | | 2016 | 2041 | 10.829(10.35-11.31) |
| Hispanic or Latino | | 2017 | 2281 | 11.378(10.90-11.85) |
| Hispanic or Latino | | 2018 | 2353 | 11.226(10.77-11.69) |
| Hispanic or Latino | | 2019 | 2476 | 11.287(10.84-11.74) |
| Hispanic or Latino | | 2020 | 3569 | 15.398(14.89-15.91) |
| Hispanic or Latino | |  | 27964 | 9.323(9.21-9.43) |

| **Supplemental Table 4**: Hypertension and Atrial Fibrillation related deaths, Stratified by region Adults in the United States, 1999 to 2020 | | |
| --- | --- | --- |
| **Census Region** | **Year** | **AAMR (95% CI)** |
| Census Region 1: Northeast | 1999 | 2.985(2.81-3.16) |
| Census Region 1: Northeast | 2000 | 8.157(7.87-8.44) |
| Census Region 1: Northeast | 2001 | 8.264(7.98-8.55) |
| Census Region 1: Northeast | 2002 | 8.863(8.57-9.16) |
| Census Region 1: Northeast | 2003 | 9.307(9.01-9.61) |
| Census Region 1: Northeast | 2004 | 9.874(9.57-10.18) |
| Census Region 1: Northeast | 2005 | 10.296(9.99-10.61) |
| Census Region 1: Northeast | 2006 | 10.36(10.05-10.67) |
| Census Region 1: Northeast | 2007 | 11.021(10.70-11.34) |
| Census Region 1: Northeast | 2008 | 11.904(11.58-12.23) |
| Census Region 1: Northeast | 2009 | 11.447(11.13-11.77) |
| Census Region 1: Northeast | 2010 | 13.706(13.36-14.05) |
| Census Region 1: Northeast | 2011 | 14.531(14.18-14.89) |
| Census Region 1: Northeast | 2012 | 15.168(14.81-15.53) |
| Census Region 1: Northeast | 2013 | 14.161(13.82-14.51) |
| Census Region 1: Northeast | 2014 | 13.854(13.51-14.19) |
| Census Region 1: Northeast | 2015 | 14.716(14.37-15.06) |
| Census Region 1: Northeast | 2016 | 14.319(13.98-14.66) |
| Census Region 1: Northeast | 2017 | 15.439(15.09-15.79) |
| Census Region 1: Northeast | 2018 | 16.161(15.81-16.52) |
| Census Region 1: Northeast | 2019 | 16.111(15.76-16.46) |
| Census Region 1: Northeast | 2020 | 20.719(20.32-21.12) |
| Census Region 2: Midwest | 1999 | 3.274(3.10-3.45) |
| Census Region 2: Midwest | 2000 | 8.811(8.53-9.09) |
| Census Region 2: Midwest | 2001 | 9.029(8.75-9.31) |
| Census Region 2: Midwest | 2002 | 10.009(9.71-10.30) |
| Census Region 2: Midwest | 2003 | 10.572(10.27-10.87) |
| Census Region 2: Midwest | 2004 | 10.902(10.60-11.21) |
| Census Region 2: Midwest | 2005 | 12.085(11.77-12.40) |
| Census Region 2: Midwest | 2006 | 12.932(12.61-13.26) |
| Census Region 2: Midwest | 2007 | 13.444(13.11-13.77) |
| Census Region 2: Midwest | 2008 | 14.402(14.06-14.74) |
| Census Region 2: Midwest | 2009 | 14.18(13.85-14.51) |
| Census Region 2: Midwest | 2010 | 14.889(14.55-15.23) |
| Census Region 2: Midwest | 2011 | 16.668(16.31-17.03) |
| Census Region 2: Midwest | 2012 | 17.253(16.89-17.61) |
| Census Region 2: Midwest | 2013 | 15.423(15.09-15.76) |
| Census Region 2: Midwest | 2014 | 16.213(15.87-16.56) |
| Census Region 2: Midwest | 2015 | 17.43(17.08-17.79) |
| Census Region 2: Midwest | 2016 | 17.713(17.36-18.07) |
| Census Region 2: Midwest | 2017 | 18.849(18.49-19.21) |
| Census Region 2: Midwest | 2018 | 19.6(19.23-19.97) |
| Census Region 2: Midwest | 2019 | 20.31(19.95-20.69) |
| Census Region 2: Midwest | 2020 | 25.4(24.99-25.81) |
| Census Region 3: South | 1999 | 2.182(2.06-2.30) |
| Census Region 3: South | 2000 | 7.474(7.26-7.69) |
| Census Region 3: South | 2001 | 7.93(7.71-8.15) |
| Census Region 3: South | 2002 | 8.848(8.62-9.08) |
| Census Region 3: South | 2003 | 9.691(9.45-9.93) |
| Census Region 3: South | 2004 | 9.667(9.43-9.91) |
| Census Region 3: South | 2005 | 11.134(10.88-11.39) |
| Census Region 3: South | 2006 | 11.281(11.03-11.53) |
| Census Region 3: South | 2007 | 12.13(11.87-12.39) |
| Census Region 3: South | 2008 | 12.41(12.16-12.68) |
| Census Region 3: South | 2009 | 12.80(12.55-13.07) |
| Census Region 3: South | 2010 | 13.676(13.41-13.94) |
| Census Region 3: South | 2011 | 14.636(14.37-14.91) |
| Census Region 3: South | 2012 | 15.693(15.42-15.97) |
| Census Region 3: South | 2013 | 14.9(14.72-15.26) |
| Census Region 3: South | 2014 | 15.133(14.87-15.40) |
| Census Region 3: South | 2015 | 16.26(15.99-16.53) |
| Census Region 3: South | 2016 | 16.82(16.55-17.09) |
| Census Region 3: South | 2017 | 18.183(17.91-18.46) |
| Census Region 3: South | 2018 | 19.204(18.92-19.48) |
| Census Region 3: South | 2019 | 20.363(20.08-20.65) |
| Census Region 3: South | 2020 | 24.238(23.93-24.55) |
| Census Region 4: West | 1999 | 3.597(3.40-3.80) |
| Census Region 4: West | 2000 | 9.522(9.20-9.85) |
| Census Region 4: West | 2001 | 10.201(9.87-10.53) |
| Census Region 4: West | 2002 | 11.353(11.01-11.70) |
| Census Region 4: West | 2003 | 11.608(11.26-11.95) |
| Census Region 4: West | 2004 | 12.57(12.21-12.93) |
| Census Region 4: West | 2005 | 13.635(13.27-14.00) |
| Census Region 4: West | 2006 | 14.421(14.05-14.79) |
| Census Region 4: West | 2007 | 14.801(14.43-15.17) |
| Census Region 4: West | 2008 | 15.555(15.18-15.93) |
| Census Region 4: West | 2009 | 15.703(15.33-16.08) |
| Census Region 4: West | 2010 | 16.862(16.48-17.24) |
| Census Region 4: West | 2011 | 18.948(18.55-19.35) |
| Census Region 4: West | 2012 | 18.874(18.48-19.27) |
| Census Region 4: West | 2013 | 17.351(16.98-17.72) |
| Census Region 4: West | 2014 | 17.448(17.08-17.81) |
| Census Region 4: West | 2015 | 18.503(18.13-18.87) |
| Census Region 4: West | 2016 | 19.568(19.19-19.94) |
| Census Region 4: West | 2017 | 20.441(20.06-20.82) |
| Census Region 4: West | 2018 | 20.784(20.41-21.16) |
| Census Region 4: West | 2019 | 21.335(20.96-21.71) |
| Census Region 4: West | 2020 | 24.886(24.48-25.29) |

| **Supplemental Table 5:** Hypertension and Atrial Fibrillation Related Age-Adjusted Mortality Rates per 10,000, Stratified by Urban-Rural Classification in the United States, 1999 to 2020 | | |  |
| --- | --- | --- | --- |
| **2013 Urbanization** | **Year** | **AAMR (95% CI)** | |
| Large Central Metro | 1999 | 2.912(2.76 - 3.06) | |
| Large Central Metro | 2000 | 8.096(7.85-8.35) | |
| Large Central Metro | 2001 | 8.753(8.49-9.01) | |
| Large Central Metro | 2002 | 9.365(9.10-9.63) | |
| Large Central Metro | 2003 | 9.876(9.60-10.15) | |
| Large Central Metro | 2004 | 10.196(9.92-10.47) | |
| Large Central Metro | 2005 | 11.114(10.83-11.40) | |
| Large Central Metro | 2006 | 11.685(11.40-11.98) | |
| Large Central Metro | 2007 | 12.051(11.76-12.34) | |
| Large Central Metro | 2008 | 12.479(12.19-12.77) | |
| Large Central Metro | 2009 | 12.691(12.40-12.98) | |
| Large Central Metro | 2010 | 14.162(13.85-14.47) | |
| Large Central Metro | 2011 | 15.268(14.95-15.58) | |
| Large Central Metro | 2012 | 15.995(15.68-16.31) | |
| Large Central Metro | 2013 | 14.485(14.19-14.79) | |
| Large Central Metro | 2014 | 14.931(14.63-15.23) | |
| Large Central Metro | 2015 | 15.814(15.51-16.12) | |
| Large Central Metro | 2016 | 15.922(15.62-16.23) | |
| Large Central Metro | 2017 | 16.399(16.09-16.70) | |
| Large Central Metro | 2018 | 16.675(16.37-16.98) | |
| Large Central Metro | 2019 | 16.931(16.63-17.23) | |
| Large Central Metro | 2020 | 21.140(20.81-21.48) | |
| Large Fringe Metro | 1999 | 2.854(2.69-3.02) | |
| Large Fringe Metro | 2000 | 7.973(7.69-8.25) | |
| Large Fringe Metro | 2001 | 8.086(7.81-8.36) | |
| Large Fringe Metro | 2002 | 8.923(8.63-9.21) | |
| Large Fringe Metro | 2003 | 9.484(9.19-9.78) | |
| Large Fringe Metro | 2004 | 9.912(9.61-10.21) | |
| Large Fringe Metro | 2005 | 10.704(10.40-11.01) | |
| Large Fringe Metro | 2006 | 10.861(10.56-11.16) | |
| Large Fringe Metro | 2007 | 11.497(11.19-11.81) | |
| Large Fringe Metro | 2008 | 11.967(11.66-12.28) | |
| Large Fringe Metro | 2009 | 11.887(11.58-12.19) | |
| Large Fringe Metro | 2010 | 13.586(13.26-13.91) | |
| Large Fringe Metro | 2011 | 14.844(14.51-15.18) | |
| Large Fringe Metro | 2012 | 15.011(14.68-15.34) | |
| Large Fringe Metro | 2013 | 14.077(13.76-14.39) | |
| Large Fringe Metro | 2014 | 14.081(13.77-14.39) | |
| Large Fringe Metro | 2015 | 15.189(14.87-15.51) | |
| Large Fringe Metro | 2016 | 15.406(15.09-15.72) | |
| Large Fringe Metro | 2017 | 16.670(16.35-17.00) | |
| Large Fringe Metro | 2018 | 17.451(17.12-17.78) | |
| Large Fringe Metro | 2019 | 18.174(17.84-18.50) | |
| Large Fringe Metro | 2020 | 22.155(21.80-22.52) | |
| Medium Metro | 1999 | 2.992(2.81-3.17) | |
| Medium Metro | 2000 | 9.017(8.71-9.32) | |
| Medium Metro | 2001 | 9.418(9.11-9.73) | |
| Medium Metro | 2002 | 10.336(10.01-10.66) | |
| Medium Metro | 2003 | 10.439(10.12-10.76) | |
| Medium Metro | 2004 | 11.286(10.96-11.62) | |
| Medium Metro | 2005 | 12.676(12.33-13.02) | |
| Medium Metro | 2006 | 13.029(12.68-13.38) | |
| Medium Metro | 2007 | 13.979(13.62-14.33) | |
| Medium Metro | 2008 | 14.703(14.34-15.06) | |
| Medium Metro | 2009 | 14.453(14.10-14.81) | |
| Medium Metro | 2010 | 15.296(14.94-15.66) | |
| Medium Metro | 2011 | 16.901(16.53-17.27) | |
| Medium Metro | 2012 | 17.500(17.13-17.88) | |
| Medium Metro | 2013 | 16.479(16.12-16.84) | |
| Medium Metro | 2014 | 16.624(16.27-16.98) | |
| Medium Metro | 2015 | 17.330(16.97-17.69) | |
| Medium Metro | 2016 | 18.358(17.99-18.72) | |
| Medium Metro | 2017 | 19.650(19.28-20.03) | |
| Medium Metro | 2018 | 20.383(20.01-20.76) | |
| Medium Metro | 2019 | 20.828(20.45-21.20) | |
| Medium Metro | 2020 | 24.455(24.05-24.86) | |
| Small Metro | 1999 | 3.102(2.84-3.37) | |
| Small Metro | 2000 | 8.600(8.16-9.04) | |
| Small Metro | 2001 | 9.006(8.57-9.45) | |
| Small Metro | 2002 | 10.114(9.65-10.58) | |
| Small Metro | 2003 | 10.924(10.45-11.40) | |
| Small Metro | 2004 | 11.157(10.68-11.64) | |
| Small Metro | 2005 | 12.508(12.01-13.01) | |
| Small Metro | 2006 | 13.465(12.95-13.98) | |
| Small Metro | 2007 | 13.520(13.01-14.03) | |
| Small Metro | 2008 | 14.684(14.16-15.21) | |
| Small Metro | 2009 | 14.706(14.19-15.23 ) | |
| Small Metro | 2010 | 15.451(14.92-15.98) | |
| Small Metro | 2011 | 18.188(17.62-18.76) | |
| Small Metro | 2012 | 18.163(17.60-18.72) | |
| Small Metro | 2013 | 16.575(16.05-17.10) | |
| Small Metro | 2014 | 16.427(15.91-16.95) | |
| Small Metro | 2015 | 17.870(17.33-18.41) | |
| Small Metro | 2016 | 19.194(18.64-19.75) | |
| Small Metro | 2017 | 20.403(19.84-20.97) | |
| Small Metro | 2018 | 21.177(20.61-21.74) | |
| Small Metro | 2019 | 22.344(21.77-22.92) | |
| Small Metro | 2020 | 26.733(26.11-27.36) | |
| Micropolitan (Nonmetro) | 1999 | 3.040(2.79-3.29) | |
| Micropolitan (Nonmetro) | 2000 | 8.919(8.49-9.35) | |
| Micropolitan (Nonmetro) | 2001 | 9.179(8.75-9.61) | |
| Micropolitan (Nonmetro) | 2002 | 10.497(10.04-10.96) | |
| Micropolitan (Nonmetro) | 2003 | 11.477(11.00-11.95) | |
| Micropolitan (Nonmetro) | 2004 | 11.734(11.25-12.22) | |
| Micropolitan (Nonmetro) | 2005 | 13.055(12.55-13.56) | |
| Micropolitan (Nonmetro) | 2006 | 13.579(13.07-14.09) | |
| Micropolitan (Nonmetro) | 2007 | 14.765(14.24-15.29) | |
| Micropolitan (Nonmetro) | 2008 | 15.215(14.68-15.75) | |
| Micropolitan (Nonmetro) | 2009 | 15.594(15.06-16.13) | |
| Micropolitan (Nonmetro) | 2010 | 16.238(15.70-16.78) | |
| Micropolitan (Nonmetro) | 2011 | 17.174(16.62-17.73) | |
| Micropolitan (Nonmetro) | 2012 | 18.384(17.82-18.95) | |
| Micropolitan (Nonmetro) | 2013 | 17.767(17.21-18.32) | |
| Micropolitan (Nonmetro) | 2014 | 18.017(17.46-18.57) | |
| Micropolitan (Nonmetro) | 2015 | 19.714(19.14-20.29) | |
| Micropolitan (Nonmetro) | 2016 | 19.288(18.73-19.85) | |
| Micropolitan (Nonmetro) | 2017 | 21.505(20.91-22.10) | |
| Micropolitan (Nonmetro) | 2018 | 22.713(22.11-23.32) | |
| Micropolitan (Nonmetro) | 2019 | 23.805(23.20-24.41) | |
| Micropolitan (Nonmetro) | 2020 | 29.91(29.23-30.59) | |
| NonCore (Nonmetro) | 1999 | 2.370(2.13-2.61) | |
| NonCore (Nonmetro) | 2000 | 7.459(7.03-7.89) | |
| NonCore (Nonmetro) | 2001 | 7.676(7.24-8.11) | |
| NonCore (Nonmetro) | 2002 | 9.135(8.66-9.61) | |
| NonCore (Nonmetro) | 2003 | 10.130(9.64-10.63) | |
| NonCore (Nonmetro) | 2004 | 10.087(9.59-10.58) | |
| NonCore (Nonmetro) | 2005 | 11.253(10.73-11.77) | |
| NonCore (Nonmetro) | 2006 | 11.475(10.95-12.00) | |
| NonCore (Nonmetro) | 2007 | 12.103(11.57-12.64) | |
| NonCore (Nonmetro) | 2008 | 13.702(13.14-14.27) | |
| NonCore (Nonmetro) | 2009 | 13.856(13.29-14.42) | |
| NonCore (Nonmetro) | 2010 | 14.574(14.0015.15) | |
| NonCore (Nonmetro) | 2011 | 15.402(14.82-15.99) | |
| NonCore (Nonmetro) | 2012 | 17.404(16.78-18.03) | |
| NonCore (Nonmetro) | 2013 | 15.809(15.22-16.40) | |
| NonCore (Nonmetro) | 2014 | 16.532(15.94-17.13) | |
| NonCore (Nonmetro) | 2015 | 18.085(17.46-18.71) | |
| NonCore (Nonmetro) | 2016 | 18.40(17.78-19.03) | |
| NonCore (Nonmetro) | 2017 | 19.946(19.30-20.59) | |
| NonCore (Nonmetro) | 2018 | 22.014(21.34-22.69) | |
| Noncore (Nonmetro) | 2019 | 24.048(23.35-24.75) | |
| Noncore (Nonmetro) | 2020 | 28.090(27.34-28.84) | |
